# Rationale, design and baseline characteristics of a 4-year (208-week) phase III trial of empagliflozin, an SGLT2 inhibitor, versus glimepiride as add-on to metformin in patients with type 2 diabetes mellitus with insufficient glycemic control

**DOI:** 10.1186/1475-2840-12-129

**Published:** 2013-09-05

**Authors:** Martin Ridderstråle, Robbyna Svaerd, Cordula Zeller, Gabriel Kim, Hans J Woerle, Uli C Broedl

**Affiliations:** 1 Steno Diabetes Center, Niels Steensens Vej 2-4, DK-2820, Gentofte, Denmark; 2 Boehringer Ingelheim AB, Liljeholmsstrand 3, 11743, Stockholm, Sweden; 3 Boehringer Ingelheim Pharma GmbH & Co. KG, Birkendorfer Straße 65, 88397, Biberach/Riß, Germany; 4 Boehringer Ingelheim Pharma GmbH & Co. KG, Binger Strasse 173, 55216, Ingelheim, Germany

**Keywords:** Blood pressure, Body weight, Empagliflozin, Glimepiride, Glycemic control, Macrovascular, Microvascular, SGLT2 inhibitor, Sulfonylurea, Type 2 diabetes

## Abstract

**Background:**

Sulfonylureas (SUs) are commonly used in the treatment of type 2 diabetes (T2DM), usually as second-line treatment after the failure of metformin. However, SUs are associated with poor durability, hypoglycemia and weight gain. Empagliflozin is a sodium glucose cotransporter 2 (SGLT2) inhibitor in development for the treatment of T2DM. In Phase II/III trials, empagliflozin reduced hyperglycemia, body weight and blood pressure, with a low incidence of hypoglycemia. The aim of this Phase III study is to compare the effects of empagliflozin and the SU glimepiride as second-line therapy in patients with T2DM inadequately controlled with metformin immediate release (IR) and diet/exercise.

**Method:**

After a 2-week placebo run-in, patients were randomized to receive empagliflozin 25 mg once daily (qd) or glimepiride 1–4 mg qd double-blind for 2 years, in addition to metformin IR. Patients who participate in the initial 2-year randomization period will be eligible for a 2-year double-blind extension. The primary endpoint is change from baseline in HbA_1c_. Secondary endpoints are change from baseline in body weight, the incidence of confirmed hypoglycemia and changes in systolic and diastolic blood pressure. Exploratory endpoints include markers of insulin secretion, body composition and responder analyses. Safety endpoints include the incidence of adverse events (AEs) (including macro- and microvascular adverse events) and changes from baseline in clinical laboratory parameters.

**Results:**

Between August 2010 and June 2011, 1549 patients were randomized and 1545 patients were treated. At baseline, mean (SD) age was 55.9 (10.4) years, HbA_1c_ was 7.92 (0.84)%, body mass index was 30.11 (5.59) kg/m^2^, systolic blood pressure was 133.5 (15.9) mmHg and diastolic blood pressure was 79.5 (9.4) mmHg.

**Discussion:**

This is the largest study to compare the efficacy and safety of an SGLT2 inhibitor with an SU in patients with T2DM inadequately controlled on metformin to date. In addition to determining the effects of these treatments on glycemic control over the long term, this study will investigate effects on beta-cell function, cardiovascular risk factors and markers of renal function/damage. The results will help to inform the choice of second-line treatment in patients with T2DM who have failed on metformin.

**Trial registration:**

Clinicaltrials.gov NCT01167881.

## Background

Type 2 diabetes mellitus (T2DM) is a chronic disease that results from a combination of insulin resistance and insulin deficiency caused by progressive beta-cell failure [1]. Treatment of T2DM should aim to control glycemia to preserve quality of life and reduce the risk of the microvascular and macrovascular complications of diabetes [2]. Metformin, the most commonly used anti-diabetes agent, is recommended as first-line therapy for patients with T2DM [2,3]. However, as glycemic control deteriorates, patients with T2DM usually require more than one anti-diabetes agent to control glycemia [4-6]. Sulfonylureas (SUs) are one of the treatment options recommended for second-line therapy of T2DM [2,3] and are commonly used in clinical practice [1,7], usually in combination with metformin.

### Sulfonylureas in the treatment of T2DM

Although initially effective in controlling hyperglycemia, SUs have low durability [1,2]. In the UK Prospective Diabetes Study (UKPDS), following an initial decline in glycosylated hemoglobin (HbA_1c_) in patients randomized to receive chlorpropamide or glibenclamide compared with patients who received dietary advice alone, a progressive increase in HbA_1c_ was observed over the next 15 years, similar to the increase that occurred in patients randomized to dietary advice alone [6]. Secondary failure rates with SUs may exceed those of other anti-diabetes agents, possibly due to increased loss of beta-cell function [1,8]. In the UKPDS, beta-cell function assessed using the homeostasis model assessment (HOMA-B) was found to be inversely proportional to failure rates with SUs [9]. In a study in newly diagnosed patients with T2DM, patients treated with an SU for up to 6 years showed a lower C-peptide response to glucagon than patients treated with insulin, suggesting a more rapid deterioration in beta-cell function and endogenous insulin production [10-12]. In a study of patients diagnosed with T2DM for more than 3 years, the duration of SU treatment was the only factor found to be independently associated with decreases in fasting C-peptide levels [13].

In addition to low durability, SUs are commonly associated with weight gain and hypoglycemia [2,14]. In patients with T2DM receiving oral anti-diabetes agents, both weight gain and hypoglycemia are independently associated with lower treatment satisfaction and lower health-related quality of life [15]. Hypoglycemic episodes lead to fear of further episodes, which may lead to patients eating more to avoid their blood glucose becoming too low, resulting in an association between hypoglycemia, fear of hypoglycemia and weight gain [15]. The magnitude of impact on quality of life has been observed to increase with the severity and frequency of hypoglycemic events experienced over a 6-month period [14] and the level of weight gain over 12 months [15]. Hypoglycemia and weight gain can also affect adherence to treatment. In a cross-sectional survey of 407 patients with T2DM, a potential weight gain of 2.3 kg over 6 months with a fictional anti-diabetes agent was associated with a 10-15% decreased likelihood of adherence compared with an agent that caused no weight gain; more than 2 episodes of mild-to-moderate hypoglycemia per month was also associated with a reduced likelihood of adherence [16]. This is important, given that adherence to medication for the treatment of T2DM is poor. In prospective studies in patients with T2DM, rates of adherence to oral anti-diabetes agents, defined as the proportion of doses taken as prescribed, have been reported to be as low as 38% [17]. Furthermore, in patients with T2DM, non-adherence to prescribed medication has been independently associated with all-cause mortality [18]. In the ACCORD study, which investigated the effect of intensive versus standard glycemic control on cardiovascular (CV) events in patients with T2DM at high CV risk, symptomatic severe hypoglycemia was associated with increased all-cause mortality [19]. The mechanisms by which hypoglycemia could precipitate a major vascular event include autonomic activation, primarily of the sympatho-adrenal system, provoking hemodynamic changes, such as increased heart rate and systolic blood pressure, increased myocardial contractility, stroke volume and cardiac output, to maintain glucose supply to the brain [20].

Microvascular complications such as albuminuria and decreasing estimated glomerular filtration rate (eGFR) are independently and continuously associated with an increased risk of CV events (CV death, non-fatal myocardial infarction, stroke) and renal events in patients with T2DM [21]. There is limited evidence that SUs reduce the microvascular complications of T2DM [6,22], but the evidence is not conclusive. In the UKPDS, after a median follow-up of 10 years, an absolute reduction of 2.8% in the incidence of microvascular endpoints was observed in patients who were randomized to intensive glucose control with an SU or insulin compared with patients randomized to dietary advice alone [6]. However, most of this benefit was attributed to a reduction in retinopathy requiring photocoagulation (absolute reduction of 3.1%). Differences were detected in progression of retinopathy, microalbuminuria, and albuminuria in favor of SU/insulin, but there were no differences in blindness, visual acuity, or renal failure [6]. This may have been because these patients were at an early stage of diabetes and so had a low risk of such complications. During the post-trial follow-up (median duration: 8.5 years), the reduced risk of microvascular events was maintained in patients who received SU/insulin compared to dietary advice alone, despite there being no difference between the groups in HbA_1c_ after 1 year [23]. In the ADVANCE study in patients with T2DM and a high risk of vascular disease, intensive glucose control provided by the SU gliclazide reduced the absolute incidence of microvascular complications compared with standard glucose control by 1.5% over a median follow-up of 5 years, a reduction that was primarily due to a reduction in new-onset microalbuminuria [22].

Most patients with T2DM die from CV-related causes [24]. The effect of SUs on macrovascular disease is unclear. In the UKPDS, intensive glucose control with SU/insulin was not associated with a reduction in macrovascular complications compared with dietary advice after a median follow-up of 10 years [6]. However, after a median follow-up of 16.8 years, significant reductions in myocardial infarction (by 15%), diabetes-related death (by 17%) and all-cause mortality (by 13%) were observed in patients randomized to receive SU/insulin compared with dietary advice alone during the intervention part of the study [23]. In the ADVANCE study, intensive glucose control provided by gliclazide did not reduce the risk of major macrovascular events in high-risk patients over a median follow-up of 5 years compared with standard glucose control [22], while in a retrospective cohort study in 5795 patients with T2DM, all-cause mortality was higher in patients receiving monotherapy with higher daily doses of first-generation SUs (adjusted hazard ratio [HR] 2.1) or glibenclamide (adjusted HR 1.3) than in patients receiving lower doses, as was mortality due to acute ischemic events (adjusted HR 1.2 and 1.4, respectively) [25]. A recent review of randomized controlled trials that evaluated the impact of SUs on CV outcomes found no increase in the incidence of CV events with SUs, but noted that the available data are limited and there have been no adequately powered trials addressing the CV safety of SUs [26].

### Empagliflozin, an SGLT2 inhibitor

Empagliflozin is a potent and selective sodium glucose cotransporter 2 (SGLT2) inhibitor [27] in development for the treatment of T2DM. SGLT2, found in the proximal tubule of the nephron, is responsible for the reabsorption of ~90% of the glucose filtered through the kidneys [28]. By blocking SGLT2, empagliflozin reduces renal glucose reabsorption, leading to excretion of glucose in the urine, thus reducing hyperglycemia in patients with T2DM [29]. In Phase II and III studies, empagliflozin improved glycemic control in patients with T2DM when used as monotherapy [30,31] or as add-on therapy [32-36]. In Phase III studies, treatment with empagliflozin was also associated with mean placebo-corrected reductions in body weight of 1.6 kg to 2.9 kg over 24 to 78 weeks [31,33-36], likely due to the loss of calories (glucose) in the urine. Further empagliflozin was associated with mean placebo-corrected reductions in systolic blood pressure of 2.1 mmHg to 4.8 mmHg over 24 to 78 weeks [31,33-36]; this may be due to a mild osmotic diuretic effect associated with urinary glucose excretion [37].

Empagliflozin is well tolerated in patients with T2DM [30-36,38,39]. As the mechanism of action of SGLT2 inhibitors is independent of the action of insulin [40,41], empagliflozin is associated with a low risk of hypoglycemia [30-36,38,39]. It is tempting to speculate that the insulin-independent mechanism of action of empagliflozin may preserve beta-cell function and provide better durability of glycemic control than SUs. In an 8-week study in Zucker Diabetic Fatty (ZDF) rats, an animal model of T2DM, treatment with empagliflozin, but not with the SU glibenclamide, preserved beta-cell mass, increased insulin levels, and improved glycemic control [42]. Furthermore, due to its body weight and blood pressure lowering properties, in addition to its effects on glycemic control, treatment with empagliflozin may have a beneficial effect on CV risk [43,44]. A large dedicated CV outcome study is underway to determine the effect of empagliflozin on CV endpoints (NCT01131676).

### Empagliflozin versus glimepiride: a 4-year phase III trial

The objective of this trial, the EMPA-REG H2H-SU™ trial, is to compare the effects of the SGLT2 inhibitor empagliflozin with the SU glimepiride given over the long term (4 years) as second-line therapy for T2DM in patients in whom metformin and diet/exercise has failed with respect to 1) glycemic control, 2) beta-cell function, 3) the CV risk factors: body weight, blood pressure and lipid levels, and 4) safety including prospectively adjudicated CV events and markers of renal function/damage. All patients have been recruited and randomized. Blinded baseline data are presented in this manuscript.

## Methods

### Patients

Adults (aged ≥ 18 years) with T2DM and insufficient glycemic control (HbA_1c_ ≥7% and ≤10%) were eligible for inclusion in the study if they had received an unchanged dose of metformin immediate release (IR) (≥1500 mg/day, or the maximum tolerated dose, or the maximum dose according to the local label) for ≥12 weeks prior to randomization and met the inclusion and exclusion criteria listed in Table 1. All participants provided written informed consent prior to screening.

**Table 1:** Key inclusion and exclusion criteria

**Inclusion criteria**	**Exclusion criteria**
Adult (aged ≥18 years) male or female patients with T2DM with insufficient glycemic control with diet, exercise and metformin IR* (≥1500 mg/day or maximum tolerated dose, or maximum dose according to local label, with dose unchanged for 12 weeks prior to randomization)	Blood glucose level >240 mg/dL (13.3 mmol/L) after an overnight fast during placebo run-in, confirmed by a 2^nd^ measurement
HbA_1c_ ≥7% and ≤10% at screening	Use of any anti-diabetes drugs other than metformin IR ≤12 weeks prior to randomization
BMI ≤45 kg/m^2^ at screening	Bariatric surgery within 2 years; treatment with anti-obesity drugs within 3 months of screening; any treatment leading to unstable body weight
Female patients: post-menopausal, or pre-menopausal and using appropriate contraception; not pregnant/breastfeeding	eGFR <60 mL/min/1.73 m^2^ (MDRD) during screening or placebo run-in
	Indication of liver disease (ALT, AST or alkaline phosphatase >3 x ULN) during screening or placebo run-in
	History of cancer within 5 years (except basal cell carcinoma)
	Acute coronary syndrome, stroke or transient ischemic attack within 3 months of informed consent
	Disorders causing unstable red blood cells; treatment with systemic steroids; change in dose of thyroid hormones within 6 weeks of screening; any uncontrolled endocrine condition (except T2DM)
	Alcohol or drug abuse within 3 months of informed consent
	Taking an investigational drug ≤ 30 days prior to receiving study drug

*Legend*: *T2DM* type 2 diabetes mellitus, *IR* immediate release, *HbA*_*1c*_ glycosylated hemoglobin, *BMI* body mass index, *eGFR* estimated glomerular filtration rate, *MDRD* Modified Diet Renal Disease formula, *ALT* alanine transaminase, *AST* aspartate transaminase, *ULN* upper limit of normal.

*One patient took metformin extended release.

### Study design

**Figure 1:**
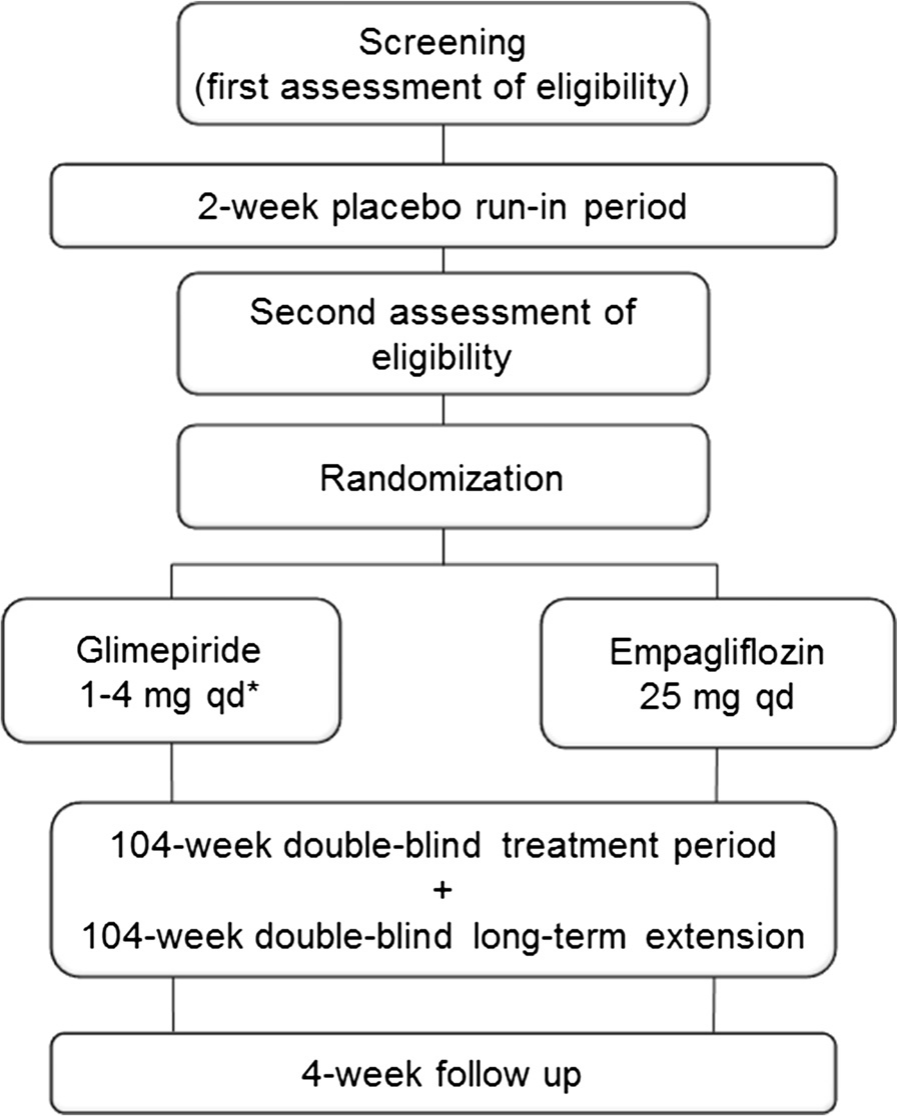
Study design. *Glimepiride was initiated at 1 mg/day, with the recommendation to uptitrate if fasting plasma glucose levels (assessed by home monitoring) were >110 mg/dL, to 2 mg/day at week 4, to 3 mg/day at week 8, and to a maximum of 4 mg/day at week 12. Uptitration can be withheld if it would place the patient at risk of hypoglycemia. Glimepiride dose can be downtitrated at any time to prevent recurrent hypoglycemia.

The protocol stated that patients were to be screened for eligibility for the study 21±7 days prior to randomization. Eligible patients were to undergo a 2-week, open-label, placebo run-in period prior to randomization, during which metformin IR was to be continued at the patient’s usual dose. Following the run-in period, patients still meeting the inclusion criteria were randomized 1:1 to receive empagliflozin 25 mg qd or glimepiride 1–4 mg qd in a double-blind, double-dummy manner for 2 years, in addition to metformin IR (Figure 1). Glimepiride was initiated at a dose of 1 mg/day, with the recommendation for uptitration if fasting plasma glucose (FPG) (assessed by home monitoring) was >110 mg/dL to 2 mg/day at week 4, to 3 mg/day at week 8, and to a maximum of 4 mg/day at week 12. Uptitration was to be withheld if it would place the patient at risk of hypoglycemia and should not take place after week 12. The glimepiride dose can be downtitrated at any time to prevent recurrent hypoglycemia. Randomization was achieved using a computer-generated random sequence communicated via a third-party interactive voice or web response system. Randomization was stratified by HbA_1c_ at screening (<8.5% and ≥8.5%), eGFR according to the Modified Diet Renal Disease (MDRD) formula (<90 mL/min/1.73 m^2^ and ≥90 mL/min/1.73 m^2^) and region (Europe/South Africa, Asia, North America, and Latin America). Patients who participate in the 2-year randomized treatment period are eligible to participate in a 2-year extension period, during which they will continue to receive the treatment allocated at randomization in a double-blind, double-dummy manner. All patients will be followed up for 4 weeks after the last dose of study drug.

Patients received diet and exercise counseling at the beginning of the placebo run-in period based on local recommendations. Patients will be reminded about the importance of following the recommended diet and exercise plan at every study visit. Rescue therapy can be initiated during the 4-year treatment period if a patient has the following confirmed blood glucose levels after an overnight fast: >240 mg/dL during weeks 1 to 12, >200 mg/dL during weeks 12 to 28 and >180 mg/dL (or HbA1c >8%) after week 28. The choice and dosage of rescue medication are at the discretion of the investigator, but must not include an SU or SGLT2 inhibitor. The use of any anti-diabetes agents other than study medication, stable background metformin IR and rescue therapy is not permitted. There are no additional restrictions on the use of concomitant medications, except for those listed in the exclusion criteria (Table 1), which will be monitored throughout the trial.

The Clinical Trial Protocol was approved by the relevant Institutional Review Boards and local Independent Ethics Committees and the trial was conducted in accordance with the Declaration of Helsinki and the International Conference on Harmonization guidelines for Good Clinical Practice.

### Endpoints

Important efficacy study endpoints are listed on the next page, and time points at which primary and key secondary efficacy parameters will be measured are shown in Table 2. The primary endpoint of this study is change from baseline in HbA_1c_. Confirmed hypoglycemic events (reported as adverse events with plasma glucose ≤3.9 mmol/L and/or requiring assistance from another person to administer carbohydrate, glucagon, or other resuscitative actions) will be evaluated as a secondary endpoint. Exploratory endpoints include change from baseline in FPG, the proportion of patients achieving HbA_1c_ <7%, effects on various biomarkers of beta-cell function including insulin, C-peptide, HOMA-B and proinsulin/insulin ratio, and the annualized slope of glycemic control (coefficient of durability). In one sub-study, 2-hour post-prandial glucose (PPG) and markers of beta-cell function and insulin resistance will be assessed in patients who undergo a mixed meal tolerance test (MTT) and in a separate sub-study, mean daily glucose (MDG) will be determined based on 8-point glucose profiles. To determine the 8-point glucose profile, patients will measure their blood glucose using a home monitoring kit at the following timepoints: after an overnight fast, 0–5 minutes before study medication and breakfast; 90 minutes after the start of breakfast; 0–5 minutes before lunch and dinner; 90 minutes after the start of lunch and dinner; 0–5 minutes before bed; and again after an overnight fast. In addition, primary, secondary and exploratory endpoints will be evaluated in a sub-group of patients with Latent Autoimmune Diabetes in Adulthood (LADA), identified by the presence at baseline of autoantibodies against insulin, islet cell cytoplasm, glutamic acid decarboxylase 65 or the intracytoplasmic domain of the tyrosine phosphatase-like protein IA-2.

**Table 2:** Timings of measurements of efficacy parameters

**HbA**_**1c**_	**At screening, randomization, after 4, 12, 28, 40, 52, 65, 78, 91, 104, 117, 130, 143, 156, 169, 182, 195 and 208 weeks of treatment**
Body weight	At screening, randomization, after 12, 28, 52, 78, 104, 130, 156, 182 and 208 weeks of treatment, and follow-up (4 weeks after treatment discontinuation)
Blood pressure	At screening, start of placebo run-in, randomization, after 4, 8, 12, 16, 28, 40, 52, 65, 78, 91, 104, 117, 130, 143, 156, 169, 182, 195 and 208 weeks of treatment, and follow-up (4 weeks after treatment discontinuation)

*Legend*: *HbA*_*1c*_ glycosylated hemoglobin.

### List of study efficacy endpoints

Primary efficacy endpoints

Change from baseline in HbA_1c_ after 52 and 104 weeks of treatment

Key secondary endpoints

Change from baseline in body weight after 52 and 104 weeks of treatment

Occurrence of confirmed hypoglycemic AEs^†^ during 52 and 104 weeks of treatment

Change from baseline in SBP and DBP after 52 and 104 weeks of treatment

Exploratory endpoints

^†^Blood glucose ≤70 mg/dL and/or assistance required.

^‡^Calculated as slope of least-squares regression line including all measurements from week 28 to last available measurement.

*Legend*: *HbA*_*1c*_ glycosylated hemoglobin, *AEs* adverse events, *SBP* systolic blood pressure, *DBP* diastolic blood pressure, *FPG* fasting plasma glucose, *MDG* mean daily glucose, *PPG* post-prandial glucose, *MTT* meal tolerance test, *DXA* Dual Energy X-ray Absorptiometry, *VAT* visceral adipose tissue, *SAT* subcutaneous adipose tissue, *MRI* magnetic resonance imaging, *BP*: blood pressure.

*Glucose control*

HbA_1c_ <7.0% or <6.5% after 52, 104 and 208 weeks of treatment

HbA_1c_ lowering by ≥0.5% after 52, 104 and 208 weeks of treatment

Change from baseline in HbA_1c_ after 208 weeks of treatment

Coefficient of durability for HbA_1c_ response^‡^

Change from baseline in FPG after 52, 104 and 208 weeks of treatment

Change from baseline in MDG (8-point) after 52, 104 and 208 weeks of treatment (sub-study)

Change from baseline in 2-h PPG after 52, 104 and 208 weeks of treatment and follow-up (4 weeks after treatment discontinuation) (sub-study)

Biomarkers of insulin secretion and resistance after 104 and 208 weeks of treatment and (in the MTT sub-study) follow-up (4 weeks after treatment discontinuation)

Confirmed hypoglycemic AEs^†^ during 208 weeks of treatment

*Weight loss*

Change from baseline in body weight of >2% and >5% after 52, 104 and 208 weeks of treatment

Change from baseline in body weight after 208 weeks of treatment and follow-up (4 weeks after treatment discontinuation)

Change from baseline in waist circumference after 52, 104 and 208 weeks of treatment and follow-up (4 weeks after treatment discontinuation)

Changes from baseline in trunk fat, limb fat, fat-free mass and total fat mass (using DXA scan) after 52, 104 and 208 weeks of treatment (sub-study)

Changes from baseline in bone mineral density and T-scores (using DXA scan) after 52, 104 and 208 weeks of treatment (sub-study)

Changes from baseline in abdominal VAT, abdominal SAT and VAT/SAT ratio (using MRI) after 52, 104 and 208 weeks of treatment (sub-study)

*Blood pressure*

Proportion of patients with BP <130/80 mmHg after 52, 104 and 208 weeks of treatment

Change from baseline in SBP and DBP after 208 weeks of treatment and follow-up (4 weeks after treatment discontinuation)

*Composite endpoints*

HbA_1c_ <7.0% or HbA_1c_ reduction ≥1.0%, no confirmed hypoglycemic AEs, and weight loss >2% after 52, 104 and 208 weeks of treatment

HbA_1c_ <6.5% or HbA_1c_ reduction ≥1.0%, no confirmed hypoglycemic AEs, and weight loss >2% after 52, 104 and 208 weeks of treatment

*Lipid profile*

Change from baseline in lipid profile after 52, 104 and 208 weeks of treatment and follow-up (4 weeks after treatment discontinuation)

Change from baseline in body weight will be evaluated as a key secondary endpoint. Exploratory endpoints include the proportion of patients with >5% reduction in body weight and change in waist circumference. The effects of empagliflozin and SU on body fat content and distribution (e.g. changes in total body fat, trunk fat, abdominal visceral adipose tissue and subcutaneous adipose tissue) will be assessed in a dedicated body composition sub-study using Dual Energy X-ray Absorptiometry (DXA) and Magnetic Resonance Imaging (MRI) scans.

Changes from baseline in systolic and diastolic blood pressure will be evaluated as key secondary endpoints. Exploratory endpoints include the proportion of patients achieving blood pressure control (<130/80 mmHg) and effects on plasma lipids. The effect of treatment on the composite endpoint of HbA_1c_ <7.0% and/or a reduction in HbA_1c_ of ≥1.0%, with no confirmed hypoglycemic events, and weight loss of >2% body weight, will be examined as an exploratory endpoint.

Safety endpoints include all AEs reported during the trial; vital signs; clinical laboratory findings; prospectively adjudicated CV events; markers of renal function/damage including changes from baseline in creatinine, eGFR and albuminuria, as well as the incidence of new-onset albuminuria; laboratory bone markers, and, in the body composition sub-study, changes from baseline in bone mineral density, as determined by DXA scan.

### Statistical analysis

The main analysis will be undertaken after 104 weeks of treatment; an interim analysis is planned after 52 weeks of treatment (Table 3). The non-inferiority of empagliflozin compared to glimepiride on the primary endpoint of change from baseline in HbA_1c_ will be tested after 52 and 104 weeks of treatment based on a one-sided significance level of 1.25%. In total, 698 patients per arm are required to provide a power of ≥95% to show non-inferiority, based on a margin of 0.3%, on the primary endpoint at 52 and 104 weeks at this significance level if the true treatment effect is 0.05% (adjusted for multiplicity related to the 52-week analysis), and the standard deviation is 1.2%.

**Table 3:** Hierarchical statistical methods

	**Analysis**	**Testing hierarchy for 52-week analysis (interim analysis)**	**Testing hierarchy for 104-week analysis (main analysis)**
1	Primary	Non-inferiority in change from baseline in HbA_1c_	Non-inferiority in change from baseline in HbA_1c_
2	Secondary	Superiority in change from baseline in body weight	Superiority in change from baseline in body weight
3		Superiority in incidence of confirmed hypoglycemic AEs	Superiority in incidence of confirmed hypoglycemic AEs
4		Superiority in change from baseline in SBP	Superiority in change from baseline in HbA_1c_
5		Superiority in change from baseline in DBP	Superiority in change from baseline in SBP
6			Superiority in change from baseline in DBP

*Legend*: *HbA*_*1c*_ glycosylated hemoglobin, *AEs* adverse events, *SBP* systolic blood pressure, *DBP* diastolic blood pressure.

The superiority of empagliflozin compared to glimepiride on the primary endpoint will be tested after 104 weeks’ treatment. Other key secondary endpoints will be tested for superiority at weeks 52 and 104, based on a significance level of 2.5% (two-sided) (Table 3). All other exploratory tests, including all analyses conducted at 208 weeks, will be 2-sided at a 5% level (no multiplicity adjustment).

The efficacy analyses will be performed on the full analysis set (FAS), i.e. all randomized patients who received ≥1 dose of study drug and had a baseline HbA_1c_ assessment, using the last observation carried forward (LOCF) methodology for imputation of missing data. Efficacy data after the first intake of rescue medication will be set to missing. For the primary endpoint, an analysis of covariance (ANCOVA) model will be used with randomized treatment, eGFR and geographical region as fixed effects and baseline HbA_1c_ as a covariate. Sensitivity analyses of the primary endpoint will be undertaken in the per-protocol set (PPS104: patients in the FAS without any important protocol violations during the first 104 weeks’ treatment, such as violating the inclusion or exclusion criteria, changing dose of background metformin IR, taking the incorrect trial medication, or compliance <80%), FAS104 completers (patients in the FAS who complete the 2-year treatment period and have HbA_1c_ values for the end of the treatment period) and PPS104 completers (FAS104 completers who do not have any important protocol violations) using the same model and the LOCF approach to missing data. Additional sensitivity analyses include a restricted maximum likelihood-based model repeated measures (MMRM) approach in observed cases in the FAS to determine the effect of empagliflozin on HbA_1c_ over time. Secondary endpoints will be analyzed using the same model as the primary endpoint, with the continuous baseline values of the endpoint being investigated included as an additional covariate. Blood pressure data after changes in antihypertensive medication will be set to missing and imputed using LOCF.

All safety endpoints will be analyzed in the treated set, i.e. all patients who received ≥1 dose of study drug and presented using descriptive statistics. Confirmed hypoglycemic events will be analyzed using Cochran-Mantel Haenszel tests stratified by baseline HbA_1c_ (<8.5% vs. ≥8.5%). Change from baseline in lipid parameters will be analyzed using ANCOVA. Time-to-event analyses (Kaplan-Meier) for onset of new microalbuminuria (albumin/creatinine ratio [ACR] ≥30 mg/g), onset of new macroalbuminuria (ACR ≥300 mg/g), and progression from microalbuminuria to macroalbuminuria groups compared using log-rank tests.

## Results

Between August 2010 and June 2011, 1549 patients across 173 sites in 23 countries were randomized to receive study drug, of whom 1545 were treated. The baseline characteristics of the treated patients are shown in Table 4. The mean (SD) age was 55.9 (10.4) years, the mean (SD) HbA_1c,_ was 7.92 (0.84)% and the mean (SD) BMI was 30.11 (5.29) kg/m^2^. Mean (SD) systolic blood pressure was 133.5 (15.9) mmHg and mean (SD) diastolic blood pressure was 79.5 (9.4) mmHg, with 68.5% of patients having uncontrolled hypertension (≥130/80 mmHg).

**Table 4:** Baseline characteristics (treated set: n=1545)

Gender, n (%)	
Male	854 (55.3)
Race, n (%)	
Caucasian	1017 (65.8)
Asian	507 (32.8)
Black/African-American	20 (1.3)
Hawaiian/Pacific Islander	1 (0.1)
Ethnicity, n (%)	
Non-Hispanic/Latino	1233 (79.8)
Hispanic/Latino	312 (20.2)
Region, n (%)	
Europe/South Africa	639 (41.4)
Asia	434 (28.1)
Latin America	276 (17.9)
North America	196 (12.7)
Age (years), mean (SD)	55.9 (10.4)
Time (years) since diagnosis of T2DM, n (%)	
≤1	172 (11.1)
>1 to 5	677 (43.8)
>5 to 10	425 (27.5)
>10	271 (17.5)
Body weight (kg), mean (SD)	82.8 (19.2)
Body mass index (kg/m^2^), mean (SD)	30.1 (5.3)
Body mass index (kg/m^2^), n (%)	
<25	243 (15.7)
25 to <30	587 (38.0)
30 to <35	434 (28.1)
≥35	281 (18.2)
Waist circumference (cm), mean (SD)	
Male	104.1 (13.0)
Female	98.8 (12.8)
HbA_1c_ (%), mean (SD)	7.9 (0.8)
HbA_1c_ (%), n (%)	
<8.5	1173 (75.9)
≥8.5	372 (24.1)
Fasting plasma glucose (mg/dL), mean (SD)	149.9 (33.9)^a^
HOMA-IR (mU/L x mmol/L), mean (SD)	6.03 (5.31)^b^
HOMA-B (mU/mmol), mean (SD)	77.0 (79.0)^c^
Cardiovascular medications, n (%)	1174 (76.0)
Antihypertensive agents	933 (60.4)
Lipid lowering agents	805 (52.1)
Acetylsalicylic acid	498 (32.2)
Systolic blood pressure (mmHg), mean (SD)	133.5 (15.9)
Diastolic blood pressure (mmHg), mean (SD)	79.5 (9.4)
Blood pressure controlled (<130/80 mmHg), n (%)	
Yes	486 (31.5)
No	1059 (68.5)
Serum lipids (mmol/L), mean (SD)	
Total cholesterol^d^	4.49 (1.01)
HDL-cholesterol^e^	1.25 (0.31)
LDL-cholesterol^f^	2.42 (0.86)
Triglycerides^e^	1.86 (1.29)
Markers of renal function and damage	
eGFR (mL/min/1.73 m^2^) according to MDRD, mean (SD)	88.0 (17.3)
eGFR (mL/min/1.73 m^2^) according to MDRD, n (%)	
≥90 (normal renal function)	633 (41.0)
60 to <90 (mild renal impairment)	877 (56.8)
30 to <60 (moderate renal impairment)	35 (2.3)
Urine albumin/creatinine ratio (mg/g), mean (SD)	40.22 (135.59)
Urine albumin/creatinine ratio (mg/g), n (%)	
<30 (normal)	1221 (79.0)
30 to <300 (microalbuminuria)	289 (18.7)
≥300 (macroalbuminuria)	35 (2.3)

*Legend:T2DM* type 2 diabetes mellitus, *HbA*_*1c*_ glycosylated hemoglobin, *HOMA-IR* homeostasis model assessment of insulin resistance, *HOMA-B* homeostasis model assessment of beta-cell function, *eGFR* estimated glomerular filtration rate, *MDRD* Modified Diet Renal Disease formula. ^a^n=1543; ^b^n=1441; ^c^n=1440; ^d^n=1518; ^e^n=1517; ^f^n=1516.

## Discussion

This head-to-head trial aims to establish comparative effectiveness between the SGLT2 inhibitor empagliflozin and the sulfonylurea glimepiride, used as second-line therapy in patients with T2DM in whom metformin and diet/exercise has failed, with regard to glycemic control, beta-cell function, diabetes-associated comorbidities such as obesity and hypertension, and safety, including macro- and renal microvascular complications, over 4 years.

This study is the largest head-to-head trial comparing an SGLT2 inhibitor with an SU to date [45,46]. As is typical of patients with T2DM, most of the patients in this study were overweight or obese and the majority had hypertension. The large number of patients enrolled in this study will enable important sub-group analyses to be performed, including analyses by the stratification factors baseline HbA_1c,_ eGFR, and region, but also analyses by baseline HOMA-IR, HOMA-B and BMI to characterize the efficacy of empagliflozin versus glimepiride in distinct subpopulations. Every effort is being made to keep patients randomized in the trial, for example, by allowing downtitration of SU to prevent recurrent hypoglycemia, by allowing patients who take rescue medication to stay in the trial, and by imposing very limited restrictions on concomitant medications. Every effort is being made to follow-up with patients who discontinue study drug.

Durability of efficacy and preservation of beta-cell function are important unmet medical needs in the treatment of patients with T2DM. This study will compare the effect of the insulin secretagogue glimepiride with empagliflozin, which has an insulin-independent mode of action, on the annualized slope of glycemic control (coefficient of durability) and biomarkers of insulin secretion over 4 years. Furthermore, the study design includes a 4-week follow-up period to assess, using a mixed MTT, whether empagliflozin truly delays or masks deterioration of beta-cell function during treatment through urinary glucose excretion.

Identification of chronic kidney disease and its progression is based on the assessment of renal function (eGFR) and markers such as albuminuria [47]. Recommendations for intensive glycemic control for prevention or delay of diabetic nephropathy are based almost exclusively on studies that have demonstrated an improvement in albuminuria [47]. In patients with T2DM and chronic kidney disease, glycemic control is recommended to be part of a multifactorial intervention strategy that includes blood pressure control [47]. Thus, empagliflozin, which lowers blood pressure and body weight as well as improving glycemic control, may offer an advantage over glimepiride in reducing the onset or deterioration of albuminuria as a surrogate marker of glomerular and tubular damage. A limitation of this study is that as the incidence of doubling of serum creatinine, end-stage renal disease or death from renal disease is expected to be low in these patients with normal renal function or mild renal impairment at baseline, this study is not powered to detect differences in hard renal outcome events.

Reduction of macrovascular complications is an important goal in the management of patients with T2DM. While this study is not powered to detect a difference in CV outcomes - assuming a yearly rate of 4-MACE (major adverse cardiac events) (CV death, non-fatal myocardial infarction, non-fatal stroke, hospitalization for unstable angina) of 1% in this low-risk population, one might expect approximately 60 adjudicated 4-MACE in a 4-year trial - exploratory comparative effectiveness data could support treatment decisions until hard evidence emerges from dedicated CV outcome trials.

## Abbreviations

ACCORD: Action to control cardiovascular risk in diabetes; ACR: Albumin/creatinine ratio; ADVANCE: Action in diabetes and vascular disease preterax and diamicron modified release controlled evaluation; AE: Adverse event; ALT: Alanine transaminase; ANCOVA: Analysis of covariance; AST: Aspartate transaminase; BP: Blood pressure; BMI: Body mass index; CV: Cardiovascular; DBP: Diastolic blood pressure; DXA: Dual Energy X-ray Absorptiometry; eGFR: Estimated glomerular filtration rate; FAS: Full analysis set; FPG: Fasting plasma glucose; HbA1c: Glycosylated hemoglobin; HOMA-B: Homeostasis model assessment of beta-cell function; HOMA-IR: Homeostasis model assessment of insulin resistance; HR: Hazard ratio; IR: Immediate release; LADA: Latent Autoimmune diabetes in adulthood; LOCF: Last observation carried forward; MACE: Major adverse cardiac events; MDG: Mean daily glucose; MDRD: Modified diet renal disease formula; MMRM: Mixed model repeated measures; MRI: Magnetic resonance imaging; MTT: Meal tolerance test; PPG: Post-prandial glucose; qd: Once daily; SAT: Subcutaneous adipose tissue; SBP: Systolic blood pressure; SGLT2: Sodium glucose cotransporter 2; SU: Sulfonylurea; T2DM: Type 2 diabetes mellitus; UKPDS: UK prospective diabetes study; ULN: Upper limit of normal; VAT: Visceral adipose tissue; ZDF: Zucker diabetic fatty [rats].

## Competing interests

This study was sponsored by Boehringer Ingelheim. RS, CZ, GK, HJW and UCB are employees of Boehringer Ingelheim. MR is head of the Patient Care Center at Steno Diabetes Center A/S, and has received honoraria for lectures in scientific meetings sponsored or arranged by Boehringer Ingelheim, Novo Nordisk A/S, Sanofi-Aventis, Novartis, Eli Lilly, Roche, Johnson & Johnson, Merck & Co, Medtronic and GlaxoSmithKline.

## Authors’ contributions

The authors meet criteria for authorship as recommended by the International Committee of Medical Journal Editors (ICMJE), were fully responsible for all content and editorial decisions, were involved at all stages of manuscript development, and have approved the final version. RS, CZ, HJW and UCB contributed to the trial design. MR contributed to the acquisition of data. All authors were involved with analysis and interpretation of data and reviewed/edited the manuscript. All authors read and approved the final manuscript.
